# P82 Implementation of a specialist surgery antimicrobial stewardship ward round

**DOI:** 10.1093/jacamr/dlag102.088

**Published:** 2026-06-26

**Authors:** Rachel Tan, Gemma Pill, Louise Dunsmure, Aaron Cordeiro, Louise Brookes, Tanya Escayo, Teig Parsons, David A Lewis, Nicola Jones

**Affiliations:** Antimicrobial Stewardship Team, Oxford University Hospitals NHS Foundation Trust, Oxford, UK; Antimicrobial Stewardship Team, Oxford University Hospitals NHS Foundation Trust, Oxford, UK; Antimicrobial Stewardship Team, Oxford University Hospitals NHS Foundation Trust, Oxford, UK; Antimicrobial Stewardship Team, Oxford University Hospitals NHS Foundation Trust, Oxford, UK; Antimicrobial Stewardship Team, Oxford University Hospitals NHS Foundation Trust, Oxford, UK; Antimicrobial Stewardship Team, Oxford University Hospitals NHS Foundation Trust, Oxford, UK; Antimicrobial Stewardship Team, Oxford University Hospitals NHS Foundation Trust, Oxford, UK; General, Renal & Perioperative Medicine, Oxford University Hospitals NHS Foundation Trust, Oxford, UK; Antimicrobial Stewardship Team, Oxford University Hospitals NHS Foundation Trust, Oxford, UK; Infectious Diseases, Oxford University Hospitals NHS Foundation Trust, Oxford, UK

## Abstract

**Background:**

Antimicrobial resistance (AMR) poses a significant threat globally and is forecasted to contribute to 1.91 million attributable deaths and 8.22 million associated deaths in 2050. Antimicrobial stewardship (AMS) initiatives have been implemented in our trust to reduce the use of broad-spectrum antibiotics. This has proven to be successful in the medical specialties and in reducing *Clostridioides difficile* infections. However, there is a lack of engagement in the surgical specialties that are heavily reliant on antimicrobials for surgical prophylaxis and treatment. We set up a specialist surgery AMS ward round to aim to improve appropriate use of antimicrobials. This comprises the ear, nose and throat (ENT), ophthalmology, oral and maxillofacial, plastic and vascular surgery specialties.

**Objectives:**

To evaluate the impact of the specialist surgery AMS ward round, in a large tertiary teaching hospital, over a 1 year period (1 April 2025 to 31 March 2026) by evaluating appropriateness of prescribing, reflected in the uptake of AMS advice by the surgical teams.

**Methods:**

We implemented a weekly specialist surgery AMS ward round in 2023. It was attended by Infection Specialist consultant and/or registrar, AMS pharmacist and AMS advanced nurse practitioner and took place on the wards with the relevant surgical team present. A peri-operative medicine consultant was also present for the vascular surgery AMS ward rounds. We extracted a list of patients who were on broad-spectrum antibiotics using the electronic prescribing system (EPR, Cerner Millenium). This included IV co-amoxiclav, third and 4^th^ generation cephalosporins, piperacillin/tazobactam, carbapenems, fluoroquinolones, clindamycin, imipenem/cilastatin, ceftazidime/avibactam, ceftolozane/tazobactam and cefepime/enmetazobactam. We documented in EPR notes, the suggested interventions made during ward rounds and evaluated the uptake of the interventions after 24 h by reviewing EPR notes.

**Results:**

We conducted 113 ward rounds, where 455 patients were reviewed, during the audited period. A total of 371 AMS recommendations were made on 270 patients. Overall, 299 (80.6%) recommendations were actioned within 24 h. The uptake of advice was highest in vascular surgery (89.7%) and lowest in oral and maxillofacial surgery (60.0%). Recommendations related to escalation of antimicrobials and interaction management were always actioned (100.0%) while advice regarding cessation of antimicrobials and requests for additional tests or samples were less frequently actioned within the specified time (74.4% and 69.4% respectively).

**Conclusions:**

Antibiotic appropriateness on specialist surgery wards improved as demonstrated by good uptake of suggested AMS advice. Notably, there was some variation in uptake of AMS advice between teams, with vascular surgery leading with nearly 90% of advice being actioned. The presence of a perioperative medicine consultant at these rounds facilitated the communication between AMS and surgery teams, as reflected in the high uptake of advice.

The importance of developing trusting relationships with surgical teams to implement sustainable AMS improvements was reinforced. Future work should explore user feedback, patient outcome measures, challenges around obtaining surgeons buy-in and creating urgency around AMS, using qualitative methods or the Capability (C), Opportunity (O), and Motivation (M) model of Behaviour.

